# Swine Enteric Coronaviruses (PEDV, TGEV, and PDCoV) Induce Divergent Interferon-Stimulated Gene Responses and Antigen Presentation in Porcine Intestinal Enteroids

**DOI:** 10.3389/fimmu.2021.826882

**Published:** 2022-01-20

**Authors:** Lingdan Yin, Xiang Liu, Dongmei Hu, Yi Luo, Guozhong Zhang, Pinghuang Liu

**Affiliations:** Key Laboratory of Animal Epidemiology of the Ministry of Agriculture, College of Veterinary Medicine, China Agricultural University, Beijing, China

**Keywords:** coronaviruses, PEDV, TGEV, PDCoV, interferons, ISG, antigen presentation

## Abstract

Swine enteric coronaviruses (SECoVs) including porcine epidemic diarrhea virus (PEDV), transmissible gastroenteritis virus (TGEV), and porcine deltacoronavirus (PDCoV), account for the majority of lethal watery diarrhea in neonatal pigs and pose significant economic and public health burdens in the world. While the three SECoVs primarily infect intestinal epithelia *in vivo* and cause similar clinical signs, there are evident discrepancies in their cellular tropism and pathogenicity. However, the underlying mechanisms to cause the differences remain unclear. Herein, we employed porcine enteroids that are a physiologically relevant model of the intestine to assess the host epithelial responses following infection with the three SECoVs (PEDV, TGEV, and PDCoV). Although SECoVs replicated similarly in jejunal enteroids, a parallel comparison of transcriptomics datasets uncovered that PEDV and TGEV infection induced similar transcriptional profiles and exhibited a more pronounced response with more differentially expressed genes (DEGs) in jejunal enteroids compared with PDCoV infection. Notably, TGEV and PDCoV induced high levels of type I and III IFNs and IFN-stimulated gene (ISG) responses, while PEDV displayed a delayed peak and elicited a much lesser extent of IFN responses. Furthermore, TGEV and PDCoV instead of PEDV elicited a substantial upregulation of antigen-presentation genes and T cell-recruiting chemokines in enteroids. Mechanistically, we demonstrated that IFNs treatment markedly elevated the expression of NOD-like receptor (NLR) family NLRC5 and major histocompatibility complex class I (MHC-I) molecules. Together, our results indicate unique and common viral strategies for manipulating the global IFN responses and antigen presentation utilized by SECoVs, which help us a better understanding of host-SECoVs interactions.

## Introduction

Swine enteric coronaviruses (SECoVs), including porcine epidemic diarrhea virus (PEDV), transmissible gastroenteritis virus (TGEV), as well as recent emergent porcine deltacoronavirus (PDCoV), have been major causative pathogens of lethal watery diarrhea in piglets (1, 2). SECoVs are highly contagious and lead to devastating consequences on the global pork industry. Alphacoronavirus PEDV and TGEV are initially reported in the 1970s and 1940s respectively and cause up to 100% mortality in neonatal piglets (3–5). Whereas PDCoV, another newly discovered deltacoronavirus in 2014, causes about 40% mortality in neonatal piglets and is less pathogenic compared with PEDV and TGEV (6). All the three SECoVs primarily infect intestinal epithelia and cause enterocyte loss, atrophy of villi, which is manifested by clinical acute, watery diarrhea and vomiting, resulting in dehydration and body weight loss (1, 2). Except for the economic impact on the swine industry, PDCoV can use aminopeptidase N (APN) from multiple species to enter cells and have the ability to infect cells of avian and mammalian species including feline and humans, which emphasizes the potential zoonotic of SECoVs and their potential spillover to humans (7–9). Therefore, a better understanding of host intestinal epithelial responses to SECoVs is critical to elucidate the pathogenesis and immunobiology of these coronaviruses and prepare for the emerging and re-emerging coronaviruses diseases in the future.

The intestinal epithelia are the largest and the first barrier of defense against viruses invading the gastrointestinal (GI) tract which can activate rapid and early cellular responses and induce the production of multiple cytokines to act as a bridge between the innate immunity and the adaptive immune responses after infection (10–12). While the clinical symptoms and lesions caused by the three SECoVs are hardly distinguishable, they also exhibit sharp contrasts in terms of overall lethality, innate immune responses of the host, and the cellular tropism (13, 14). Emerging evidence shows that PEDV, unlike TGEV and PDCoV, does not elicit robust type I interferon (IFNs-α/β) and type III IFN-lambda (IFN-L) and exploits various strategies to evade the IFN responses in different cellular models (15–20). However, there are no reports of parallel comparison of the cellular responses following the three SECoVs infections in primary small intestinal epithelia, the main intestinal segmental targets *in vivo* (1). A comprehensive clarification of the similarities and differences of innate immunity in small intestinal epithelia elicited by different SECoVs helps identify both conserved and pathogen-unique host responses, elucidate the underlying mechanisms of overlapping but unique pathogenesis, as well as develop novel therapies against swine enteric coronavirus disease (SECD).

The intestinal enteroids derived from porcine crypt stem cells closely resemble the original epithelial tissue in terms of cellular complexity and expressed genes, and are an excellent *ex vivo* model to explore the cellular responses to SECoVs infection (18, 20). In this study, we performed and compared the transcriptomics of porcine jejunal enteroids infected with the three SECoVs (PEDV, TGEV, and PDCoV) by RNA-Seq. The systematic analysis of transcriptional landscape demonstrated distinct approaches utilized by PEDV, TGEV, and PDCoV to manipulate the host immunity. Dividing differentially expressed genes by function, we found that TGEV and PDCoV substantially elicited the IFN and IFN-stimulated gene (ISG) responses, but to a much lesser extent in the PEDV scenario. In line with the IFN responses, PEDV, unlike TGEV and PDCoV, did not robustly upregulate the expression of major histocompatibility complex class I (MHC-I) [known as the swine leukocyte antigen class I (SLA-I) in pigs] and its associated antigen presentation genes. Mechanistic studies revealed that IFNs stimulation prominently increased the expression of NOD-like receptor (NLR) family NLRC5 and SLA-I molecules. Collectively, our data highlight the unique and overlapping innate immunity of primary intestinal epithelia in response to different SECoVs infection, which provides important insights into the host-SECoVs interactions and pathogenesis.

## Materials and Methods

### Cells and Viruses

Porcine intestinal epithelial cell lines IPEC-J2 were cultured in Dulbecco’s Modified Eagle’s Medium Nutrient Mixture F-12 (DMEM-F12) containing 100 U/mL penicillin, 100 μg/mL streptomycin, and 10% heat-inactivated fetal bovine serum (FBS) (Gibco, USA). The PDCoV NH strain (GenBank Accession No. KU981062.1) and TGEV AHHF strain (GenBank Accession No. KX499468.1) were grown and titrated as previously described (18, 21). The PEDV-JMS strain was isolated and maintained in our laboratory, and the PEDV stock was prepared and titrated as previously described (20).

### Porcine Intestinal Two-Dimensional (2D) Enteroid Monolayer Culture

Porcine intestinal crypts were isolated and cultured following previously described instructions (18, 20). In brief, porcine jejunum tissues were obtained from specific-pathogen-free (SPF) 2-day-old piglets, cut into 2-mm segments and washed using ice-cold phosphate-buffered saline (PBS) supplemented with 1% penicillin-streptomycin until the supernatant was clear. The intestinal pieces were then dissociated with Gentle cell dissociation reagent (Stemcell, Canada) for 30 min at room temperature (RT), suspended in ice-cold PBS with 0.1% bovine serum albumin (BSA), and passed through a 70-μm cell strainer to harvest the crypts. Approximately 50 isolated crypts were plated in each well of a 48-well plate onto a thin layer of Matrigel (BD Biosciences, USA) and grown in IntestiCult organoid growth medium (Stemcell, Canada) to differentiate 3D enteroids. To better model viral infections of the apical surface of enteroids, we established 2D monolayer enteroids from 3D enteroids as previously described (18). Briefly, 3D jejunal enteroids after 7 days of growth were washed with ice-cold DMEM-F12, dissociated with 0.25% trypsin (Gibco, USA) for 10 min at 37°C, and then the trypsin was inactivated by DMEM-F12 containing 10% (vol/vol) FBS. Following dissociation of the cells by repeated pipetting, the cells were pelleted for 5 min at 800 × g, suspended in IntestiCult Organoid Growth Medium, and seeded in a 96-well plate precoated with Matrigel at 50 enteroids per well. The 2D enteroid monolayers were ready for use in experiments after differentiation for 3 days. All the protocols related to the animal experiments were approved by the Laboratory Animal Ethical Committee of China Agricultural University.

### Viral Infections

SECoVs infections were carried out at an MOI of 5 (PEDV), MOI of 1 (TGEV), and MOI of 2 (PDCoV). 2D enteroid monolayer cultures were respectively infected with PEDV, TGEV, and PDCoV at the indicated MOI or mock infected with DMEM-F12 and incubated at 37°C for 2 h. Enteroid monolayers were then washed and grown in IntestiCult Organoid Growth Medium. Samples were harvested at the time points post infection indicated on the figures.

### RNA Extraction and Reverse Transcriptase Quantitative PCR

Total cellular RNA was extracted using a Simply P total RNA extraction kit (BioFlux, China) according to the protocols of the manufacturer. The cDNA was synthesized from 1 μg of total RNA by reverse transcription using a PrimeScript II first-strand cDNA synthesis kit (TaKaRa, Japan) following the manufacturer’s instructions, and was subjected to qPCR performed in triplicate using SYBR green PCR mix (Life Technologies, USA) on a LightCycler 480 II system (Roche, Switzerland). The cellular glyceraldehyde-3-phosphate dehydrogenase (GAPDH) expression was quantified as the internal control. The 2^-ΔΔCT^ method was used to calculate the relative expression of target genes compared to GAPDH. The primers used for qPCR are listed in **Table 1**.

**Table 1:** Sequences of primers for qPCR.

Primer	Sequences (5’-3’)
PEDV-qPCR-F	CACTTATTGGCAGGCTCTTT
PEDV-qPCR-R	CCATTGAGAAAAGAAAGTGTAGTAG
TGEV-qPCR-F	GCTTGATGAATTGAGTGCTGATG
TGEV-qPCR-R	CCTAACCTCGGCTTGTCTGG
PDCoV-qPCR-F	AGCAACCACTCGTGTTACTTG
PDCoV-qPCR-R	CAACTCTGAAACCTTGAGCTG
RIG-I-qPCR-F	GGCTGAAGCCACAGAATA
RIG-I-qPCR-R	TCAGTGGTCCGTAATTCC
NLRC5-qPCR-F	TCCAAACAAGTGCGATGA
NLRC5-qPCR-R	TGAGCCAGTTCCCAGATT
IFN-β-qPCR-F	AGCACTGGCTGGAATGAAAC
IFN-β-qPCR-R	TCCAGGATTGTCTCCAGGTC
IFN-L1-qPCR-F	CCACGTCGAACTTCAGGCTT
IFN-L1-qPCR-R	ATGTGCAAGTCTCCACTGGT
IFNAR1-qPCR-F	ACATCACCTGCCTTCACCAG
IFNAR1-qPCR-R	CATGGAGCCACTGAGCTTGA
OAS1-qPCR-F	GAGTTTTCCACCTGCTTCACG
OAS1-qPCR-R	AAATCTGTTTTCCCGCTTCCT
OASL-qPCR-F	TCCCTGGGAAGAATGTGCAG
OASL-qPCR-R	CCCTGGCAAGAGCATAGTGT
RSAD2-qPCR-F	AAGCAGAGCAGTTTGTTATCAGC
RSAD2-qPCR-R	TTCCGCCCGTTTCTACAGT
IFIT2-qPCR-F	TGAAATGTGTGGGAAAAGAGA
IFIT2-qPCR-R	CAGAGGCAGGCGAGATAGGAG
IFITM3-qPCR-F	GTCGTCTGGTCCCTGTTCAAC
IFITM3-qPCR-R	GAGTAGGCGAAAGCCACGAA
ISG15-qPCR-F	AGCATGGTCCTGTTGATGGTG
ISG15-qPCR-R	CAGAAATGGTCAGCTTGCACG
β2M-qPCR -F	CCTGTCTTTCAGCAAGGA
β2M-qPCR -R	CGGTTAGTGGTCTCGATC
PSMB9-qPCR-F	GAGAAGTCCACACTGGGA
PSMB9-qPCR-R	CGCACAGTAGATTCGATG
TAP1-qPCR-F	ACGGGGACTGTGTCTCTT
TAP1-qPCR-R	GAGATTCCTGCACCTGTG
SLA-1-qPCR-F	GTGGCTGGAGTTGTGATC
SLA-1-qPCR -R	ACCCTTGGTAAGGGACAC
SLA-2-qPCR -F	AGGGAGAGAGGAGCTACC
SLA-2-qPCR -R	ATGTGTCTTTGGAGGCTC
SLA-3-qPCR -F	CACAGACTTTCCGAGTGA
SLA-3-qPCR -R	TAGGCGTCCTGACTGTAC
GAPDH-qPCR-F	CCTTCCGTGTCCCTACTGCCAAC
GAPDH-qPCR-R	GACGCCTGCTTCACCACCTTCT

### Immunofluorescence Assay

Enteroid monolayers grown in 96-well plates were respectively infected with the three SECoVs for 24 h. Enteroid monolayers were fixed with 4% paraformaldehyde (PFA) for 30 min at RT, permeabilized with PBS containing 0.2% Triton X-100 for 20 min, and then blocked with 5% FBS and 5% skim milk (Sigma-Aldrich, USA) diluted in PBS at 37°C for 2 h. The cells were respectively incubated with nucleocapsid (N) antibody of PEDV, TGEV, or PDCoV overnight at 4°C, and then labeled with Alexa Fluor 546 goat anti-mouse IgG antibody (Thermo Fisher Scientific, USA) at 37°C for 1 h. DAPI (4’,6-diamidino-2-phenylindole) was used to stain nuclear DNA. The cells were imaged using an Evos FL Auto2 fluorescence microscope.

### RNA-Seq

Enteroid monolayers were respectively infected with PEDV (MOI = 5), TGEV (MOI = 1), and PDCoV (MOI = 2), or mock-infected with DMEM-F12 for 24 h and were harvested for RNA-Seq as previously described (18). Briefly, total RNA was extracted from infected and uninfected enteroids using the TRIzol reagent according to standard protocols. RNA purity and integrity were assessed by an Agilent 2100 Bioanalyzer (Agilent Technologies), a NanoDrop instrument (Thermo Fisher Scientific), and 1% agarose gel electrophoresis, and a total amount of 3 μg RNA per sample was used for library preparation using the NEBNext^®^ UltraTM RNA Library Prep Kit for Illumina^®^ (NEB, USA) following manufacturer’s recommendations. The library preparations were sequenced on the Illumina HiSeq 4000 platform (Illumina). All sequences were processed and aligned to the *Sus scrofa* reference genome. The Deseq2 R package was used to determine differentially expressed genes at a significance cutoff of *P* value < 0.05. The differentially expressed genes (DEGs) were selected based on the screening criteria that | log_2_ (fold change) | > 1 and false discovery rate (FDR) < 0.05.

### IFNs Treatment

To assess the ability of IFNs to induce the expression of MHC molecules, IPEC-J2 cells grown to 90% confluence on coverslips in 24-well plates were respectively stimulated with IFN-β, IFN-γ, and IFN-L at the indicated concentrations for 24 h at 37°C. The cells were then collected for RT-qPCR analysis.

### Statistical Analysis

All data were obtained from at least three independent experiments as indicated in the figure legends or as detailed and are presented as the means ± the standard errors of the mean (SEMs). Comparison between groups was performed with the Student’s *t* test using GraphPad Prism version 7 (GraphPad Software, Inc.). *P*-values < 0.05 were considered statistically significant.

## Results

### Porcine Intestinal Enteroids Support Robust Infections of PEDV, TGEV, and PDCoV

**Figure 1:**
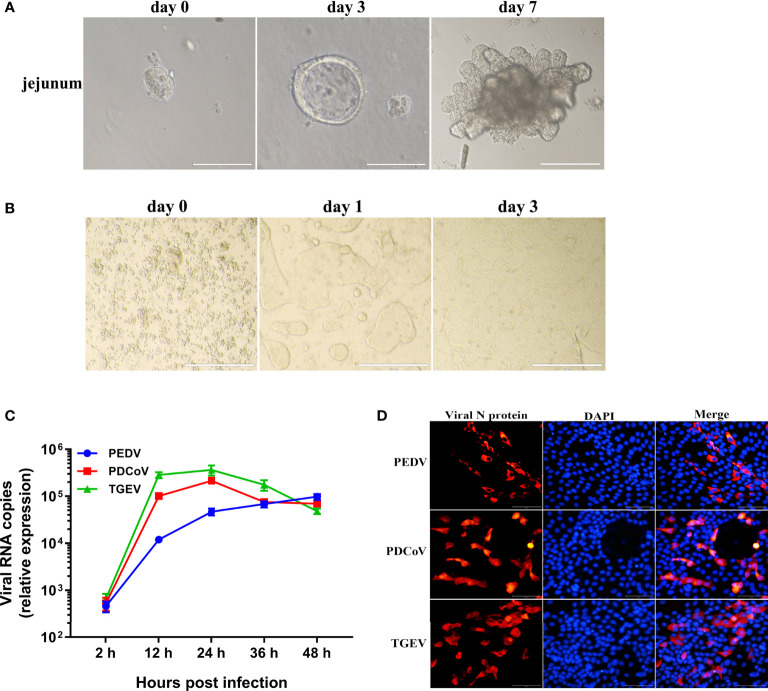
Porcine enteroids are susceptible to PEDV, TGEV, and PDCoV infection. **(A)** Representative images of the proliferation and differentiation of porcine jejunal enteroids derived from crypt stem cells of jejunum. The isolated intestinal crypts cultured in proliferation medium mixed with Matrigel gradually formed 3D budding organ-like structures on day 7. Bar = 100 μm (day 0, 3) or 200 μm (day 7). **(B)** Representative images of 2D jejunal enteroid monolayers generated from differentiated 3D enteroids. After culturing for 7 days, 3D jejunal enteroids were dissociated into single cells and seeded onto 96-well plates precoated with Matrigel to differentiate for 3 days. **(C)** The kinetic curves of SECoVs replication in jejunal enteroids. Jejunal enteroid monolayers were respectively infected with PEDV (MOI = 5), TGEV (MOI = 1), or PDCoV (MOI = 2). The kinetics of SECoVs replication at the indicated time points post infection was determined by RT-qPCR. Each data bar represents the means ± SEMs (n = 3). **(D)** Examination of SECoVs infection in jejunal enteroids by IFA. Jejunal enteroid monolayers were respectively infected with PEDV (MOI = 5), TGEV (MOI = 1), or PDCoV (MOI = 2) for 24 h, and then were harvested for staining the N protein of SECoVs using the corresponding mouse anti-N monoclonal antibodies (red). DAPI-stained nuclei were shown in blue. Bar = 100 μm.

We and others have previously demonstrated that stem cell-derived enteroids from porcine small intestines are permissive to SECoVs and can model the events associated with SECoVs infections *in vivo* (18, 20, 22, 23). Given that the detailed host responses of primary small intestinal epithelia to SECoVs infection remain unclear, we therefore utilized porcine jejunal enteroids as an *ex vivo* infection model to study the interactions of host and SECoVs infection. To this end, we firstly isolated crypt stem cells from the jejunum of 2-day-old piglets, which were cultured in a proliferation medium mixed with Matrigel to differentiate into three-dimensional (3D) jejunal enteroids as previously described (18, 20). Following a culturing period of 7 days, intestinal stem cells proliferated and differentiated, budding into enteroids containing villus-like structures (**Figure 1A**).

Since it is hard for pathogens to access the apical surface orientated inwards of enteroids, infection of 3D enteroids is challenging. To better model enteric virus infections of the apical surface of enteroids, we next established 2D enteroid monolayers from differentiated 3D jejunal enteroids using previous descriptions (18, 20). After differentiation and expansion, the seeded cells gradually grew into large, contiguous sheets of the epithelium (**Figure 1B**). We subsequently monitored the replication of PEDV, TGEV, and PDCoV propagations in 2D jejunal enteroids by detecting the expression of viral genomes and N proteins. Consistent with previously reported results (18, 20, 22), jejunal enteroids were susceptible to all the three SECoVs (**Figures 1C, D**). The kinetics of TGEV and PDCoV infection displayed a similar pattern, the genomes of TGEV and PDCoV both reached a peak at 24 h post infection (hpi), with a 552-fold and 403-fold increase compared with the number at 2 hpi, respectively, and maintained a plateau after 24 hpi, whereas PEDV genomes peaked at 48 hpi and increased by 212-fold, showing a slightly lower level of replication than that of TGEV and PDCoV (**Figure 1C**) though at a higher multiplicity of infection (MOI). The successful SECoVs infections in jejunal enteroids were further confirmed using immunofluorescence assay (IFA) for viral N protein (**Figure 1D**). Taken together, these data show that porcine enteroids are susceptible to all the three SECoVs infections and are good *in vitro* infection models of SECoVs.

### Different SECoVs Infection Induces Both Unique and Overlapping Transcriptional Profiles in Porcine Enteroids

**Figure 2:**
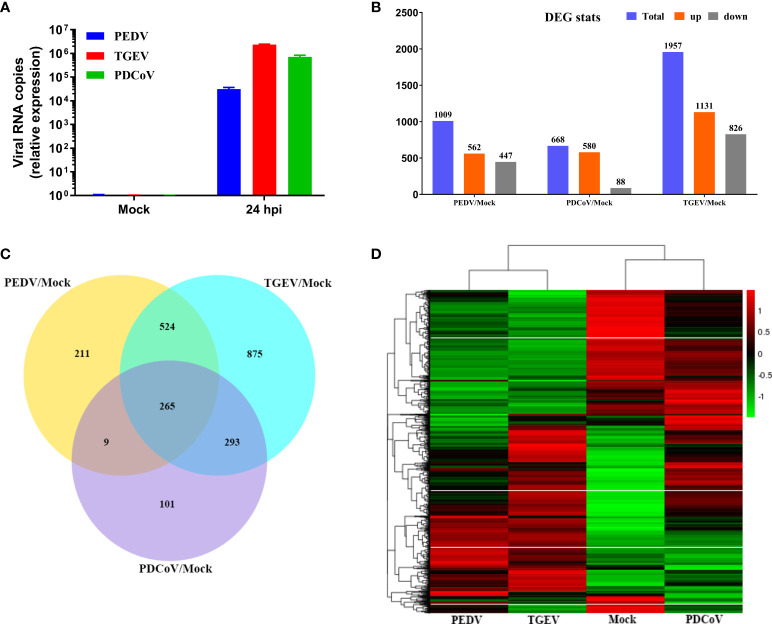
Unique and overlapping transcriptional profiles in porcine enteroids following different SECoVs infection. **(A)** Confirmation of SECoVs infection in jejunal enteroids by RT-qPCR. Jejunal enteroid monolayers were respectively infected with PEDV (MOI = 5), TGEV (MOI = 1), or PDCoV (MOI = 2) for 24 h, and collected for quantifying the viral genomes of SECoVs. Each data bar represents the means ± SEMs (n = 3). **(B)** The statistical graph results of differential expressed genes of jejunal enteroids after infection with PEDV, TGEV, or PDCoV. Jejunal enteroid monolayers isolated from three individual SPF 2-day-old piglets were mock-infected or infected with PEDV (MOI = 5), TGEV (MOI = 1), or PDCoV (MOI = 2) for 24 h. Then, the total RNA isolated from enteroids was used for RNA-Seq. **(C)** Venn diagram of common differentially expressed genes of jejunal enteroids elicited by PEDV, TGEV, or PDCoV. **(D)** Heat map of differentially expressed genes in jejunal enteroids infected with PEDV, TGEV, or PDCoV. Data are based on log(RPKM) values (n = 3).

To characterize the global cellular responses of primary intestinal epithelia to the three SECoVs infections, we performed transcriptional profiling of jejunal enteroids infected with or without PEDV, TGEV, or PDCoV by high-throughput RNA sequencing (RNA-Seq). Initially, we evaluated viral genomes levels using reverse transcriptase quantitative PCR (RT-qPCR), revealing an approximately 4.48 log_10_ increase of PEDV genomes, 6.37 log_10_ increase of TGEV genomes, and 5.85 log_10_ increase of PDCoV genomes at 24 hpi compared with mock-infected control (**Figure 2A**). We next performed transcriptional expression analysis after the three SECoVs infections of jejunal enteroids. Compared with mock-infected control, PEDV infection led to a total of 1009 DEGs, including 562 upregulated and 447 downregulated genes, and TGEV infection upregulated 1131 genes and downregulated 826 genes among the total of 1957 DEGs; whereas PDCoV infection caused a less number of DEGs (668 genes in total), including 580 upregulated and 88 downregulated genes (**Figure 2B**), which was consistent with the results that PDCoV infection elicits a less number of DEGs in porcine intestinal epithelial cells (IPEC-J2) than in human intestinal epithelial cells (HIEC) (24), suggesting a distinct transcriptional profile of enteroids after different SECoVs infections, especially the alphacoronavirus PEDV and TGEV compared with PDCoV. The Venn diagram further showed that PEDV, TGEV, and PDCoV infection yielded diverse DEGs profiles in comparison with mock-infected control, with only 265 shared genes among these three SECoVs-infected enteroids (**Figure 2C**). This was consistent with the heat map results that the transcriptional profiles of jejunal enteroids after PEDV and TGEV infection displayed a similar pattern, even though the differential expression of TGEV-infected enteroids was more significant than PEDV, whereas PDCoV infection induced distinct expression profiles from those of PEDV and TGEV, but a lesser differential degree compared with TGEV (**Figure 2D**). Altogether, these data demonstrate that the transcriptional expression profiles of jejunal enteroids induced by the three SECoVs infections not only exhibit a virus-specific manner but also share some similarities.

### PEDV and TGEV, Not PDCoV, Exert a Similar Effect on the Biological Function of Porcine Enteroids

**Figure 3:**
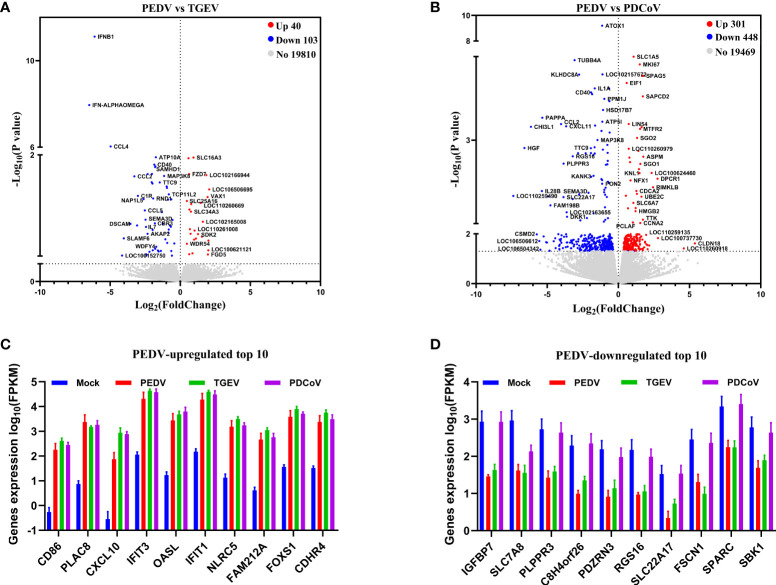
PEDV and TGEV, not PDCoV, exert a similar effect on the biological function of porcine enteroids. **(A, B)** Volcano plot of all expressed host genes of PEDV-infected enteroids relative to TGEV-infected enteroids **(A)** and PDCoV-infected enteroids **(B)**. The y-axis shows the -log_10_ transformed P-value, and the x-axis shows the log_2_ fold change in normalized expression; the red, blue, and gray points represent the significantly upregulated, downregulated, and non-significant differentially expressed genes, respectively (*P* < 0.05). **(C, D)** The transcriptional expressions of PEDV-elicited top 10 significantly upregulated **(C)** or downregulated **(D)** genes in the three SECoVs-infected enteroids were evaluated based on log(RPKM) values (n = 3).

**Figure 4:**
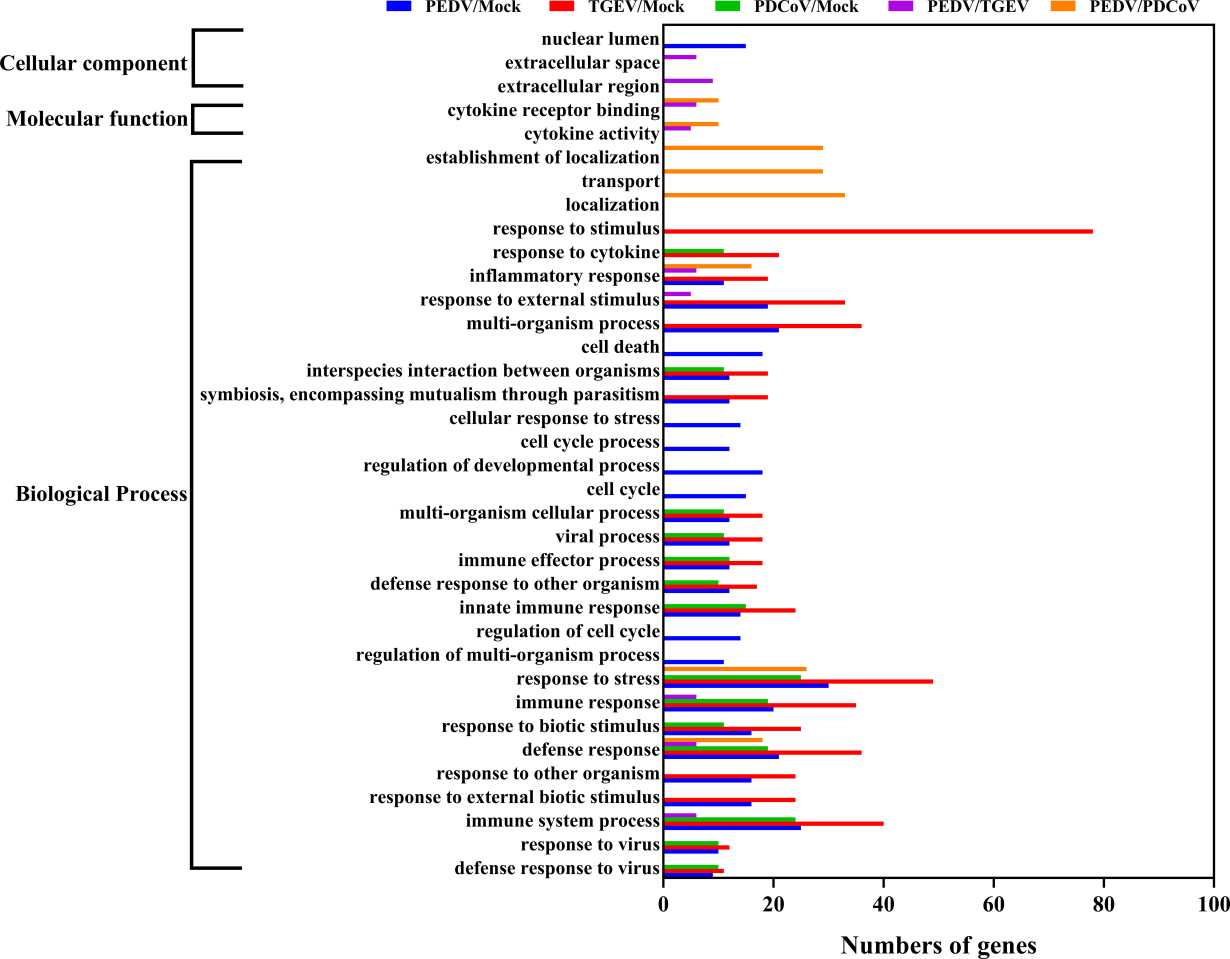
GO enrichment analysis of PEDV, TGEV, or PDCoV targets.

To further distinguish the functional changes of enteroids in response to PEDV, TGEV, and PDCoV infection, we initially compared the PEDV-elicited DEGs with those of TGEV and PDCoV, respectively, and to be graphed in a volcano plot. The results of the volcano plot indicated that compared with the TGEV-infected groups, PEDV infection only upregulated 40 genes and downregulated 103 genes, while PEDV infection induced 301 upregulated genes and 448 downregulated genes compared with the PDCoV-infected groups (**Figures 3A, B**). We then selected top 10 PEDV significantly up- and down-regulated genes and further analyzed the transcriptional levels of these genes in the three SECoVs-infected enteroids, respectively. Both TGEV and PDCoV infection robustly upregulated the expression of the top 10 PEDV-enhanced genes compared with mock-infected control (**Figure 3C**). Unexpectedly, we found that TGEV infection, but not PDCoV infection, obviously reduced the expression of the top 10 PEDV-downregulated genes (**Figure 3D**), indicating a more significant difference between PEDV and PDCoV compared with TGEV. To comprehensively clarify the functional consequences of the gene profiles elicited by the three SECoVs, we applied a gene ontology (GO) enrichment analysis to our RNA-Seq differential expression data. We revealed that most of the DEGs induced by PEDV and TGEV were enriched in the biological process, while rare of which were enriched in molecular function and cellular component, but the numbers of DEGs induced by PEDV were greatly less than those induced by TGEV (**Figure 4**). However, only a few DEGs caused by PDCoV were enriched in the biological process (**Figure 4**), suggesting that PEDV and TGEV caused more cellular functional changes than PDCoV. In addition, we observed that the DEGs elicited by all the three SECoVs infections were largely associated with response to stress, immune system process, defense response, immune response, and innate immune response (**Figure 4**), indicating that all the three SECoVs infections might modify the immune responses of host intestinal epithelia. Overall, these results demonstrate that the DEGs induced by PEDV, TGEV, and PDCoV not only play a diverse role in the regulation of biological processes but also involve in mediating some common functions, especially in relation to the cellular immune responses.

### TGEV and PDCoV Infection Elicits a Stronger IFN/ISG Response in Enteroids Than PEDV

**Figure 5:**
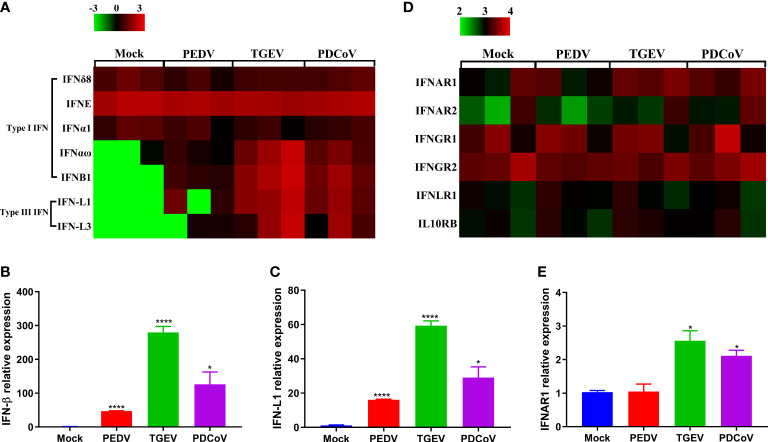
PEDV infection displays a weaker induction of IFN responses in enteroids than TGEV and PDCoV. **(A, D)** Heat map of IFNs and IFN receptors expression in enteroids after SECoVs infection. Jejunal enteroid monolayers were mock-infected or infected with PEDV, TGEV, or PDCoV for 24 h. The differential expression profiles of IFNs **(A)** and IFN receptors **(D)** were analyzed by RNA-Seq. Data are based on log(RPKM) values (n = 3). **(B, C, E)**. Confirmation of IFNs and IFNAR1 expression in enteroids after SECoVs infection by RT-qPCR. Monolayers of jejunal enteroids were infected with PEDV, TGEV, or PDCoV for 24 h. The mRNA expressions of IFN-β **(B)**, IFN-L1**(C)**, and IFNAR1 **(E)** were assessed by RT-qPCR. The results are presented as the means ± SEMs (n = 3). The differences between groups were compared using the Student’s *t* test. Error bars denote the deviations, and the *p* values are expressed as follows: *p <0.05, and ****p <0.001.

Upon viral infections, the host innate immune system may be triggered and quickly induces the production of IFNs, which further bind to their corresponding receptors and lead to the induction of a broad range of IFN-stimulated genes (ISGs), thus establishing the strong antiviral state (25, 26). As we observed above that the functional changes caused by all the three SECoVs were closely associated with innate immunity (**Figure 4**), we, therefore, focused our analysis on the IFN and ISG responses in jejunal enteroids after infection with PEDV, TGEV, and PDCoV. We initially analyzed the transcriptional profiles of type I and III IFNs in jejunal enteroids after infection with SECoVs, and the results showed that the levels of type I IFNB1 and type III IFNs (IFN-L1 and IFN-L3) in enteroids were notably upregulated by TGEV infection, followed by PDCoV; whereas PEDV infection only induced a minor expression of type I and III IFNs (**Figure 5A**). Furthermore, the expressions of other type I IFNs exhibited no obvious alteration after infection (**Figure 5A**). Consistent with the results of RNA-Seq, the mRNA levels of IFN-β and IFN-L1 in jejunal enteroids detected by RT-qPCR were remarkably elevated by TGEV, followed by PDCoV and PEDV (**Figures 5B, C**). However, TGEV and PDCoV replicated efficiently in enteroids despite high quantities of type I and III IFNs (**Figure 2**), demonstrating that unlike PEDV delaying IFNs production, they may have evolved different mechanisms to evade the IFN responses. Some viruses have been reported to evade immune responses by downregulating IFN receptors expression (27–29). To explore whether SECoVs infection affects IFN receptors expression, we next compared the receptor expression of the three types of IFNs, including type I IFN receptors (IFNAR1 and IFNAR2), type II IFN receptors (IFNGR1 and IFNGR2), and type III IFN receptors (IL10RB and IFNLR1) (30). We uncovered that the expression levels of IFN receptors were not inhibited; instead, the expression of IFNAR1 was significantly increased by TGEV infection, followed by PDCoV infection, whereas PEDV infection had no impact on the expression of IFN receptors (**Figure 5D**). The elevation of IFNAR1 was further validated by RT-qPCR, which was in accord with the data of RNA-Seq (**Figure 5E**), indicating that SECoVs do not evade the IFN responses by downregulating the expression of IFN receptors. Together, these results suggest that the induction of IFN responses in enteroids by the three SECoVs is significantly differential, and the ability of TGEV and PDCoV to activate IFN signals is superior to PEDV.

**Figure 6:**
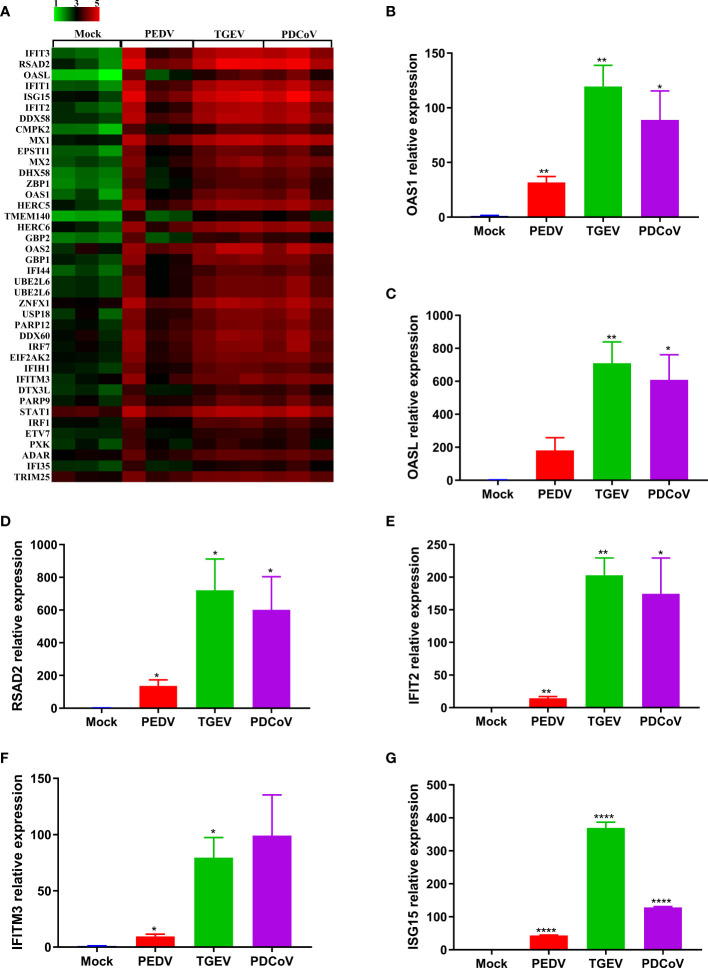
PEDV infection elicits a poorer ISG response in enteroids than TGEV and PDCoV. **(A)** Heat map of robustly upregulated ISGs expression in enteroids following SECoVs infection. Jejunal enteroid monolayers infected with PEDV, TGEV, or PDCoV, and collected at 24 hpi for the analysis of ISGs expression profiles by RNA-Seq. Data are based on log(RPKM) values (n = 3). **(B–G)** Verification of ISGs expression in enteroids after SECoVs infection by RT-qPCR. The mRNA levels of OAS1 **(B)**, OASL **(C)**, RSAD2 **(D)**, IFIT2 **(E)**, IFIM3 **(F)**, and ISG15 **(G)** in jejunal enteroids following PEDV, TGEV, and PDCoV were measured by RT-qPCR. The results are presented as the means ± SEMs (n = 3). The differences between groups were compared using the Student’s *t* test. Error bars denote the deviations, and the *p* values are expressed as follows: *p <0.05, **p <0.01, and ****p <0.001.

To further confirm the differential ability to elicit the IFN responses by the three SECoVs, we evaluated the expression profiles of ISG responses in jejunal enteroids following PEDV, TGEV, and PDCoV infection. Consistent with the IFNs production, TGEV infection led to the highest upregulation of ISGs expression, followed by PDCoV; whereas PEDV infection induced the lowest upregulation of ISGs (**Figure 6A**). Moreover, we selected several typical ISG representatives, including 2’-5’-oligoadenylate synthetase 1 (OAS1), 2’-5’-oligoadenylate synthetase-like protein (OASL), radical S-adenosyl methionine domain-containing 2 (RSAD2), interferon-induced protein with tetratricopeptide repeats 2 (IFIT2), interferon-induced transmembrane protein 3 (IFITM3), and interferon-stimulated gene 15 (ISG15), and verified their mRNA levels in enteroids following SECoVs infection by RT-qPCR. In agreement with the results of RNA-Seq, the mRNA levels of all the ISGs were prominently increased in jejunal enteroids after the three SECoVs infections, and the elevation of ISGs was the most remarkable in TGEV-infected enteroids, followed by PDCoV-infected enteroids and PEDV-infected enteroids (**Figures 6B**–**G**). Collectively, all the three SECoVs infections in enteroids activate the IFN signaling and ISG responses, but the ability of each of SECoVs to induce the antiviral immune responses is markedly differential.

### The Differential Ability of SECoVs to Induce IFN/ISG Responses Is Related to Their Varied Regulation of PRRs and NLRs Expression in Enteroids

**Figure 7:**
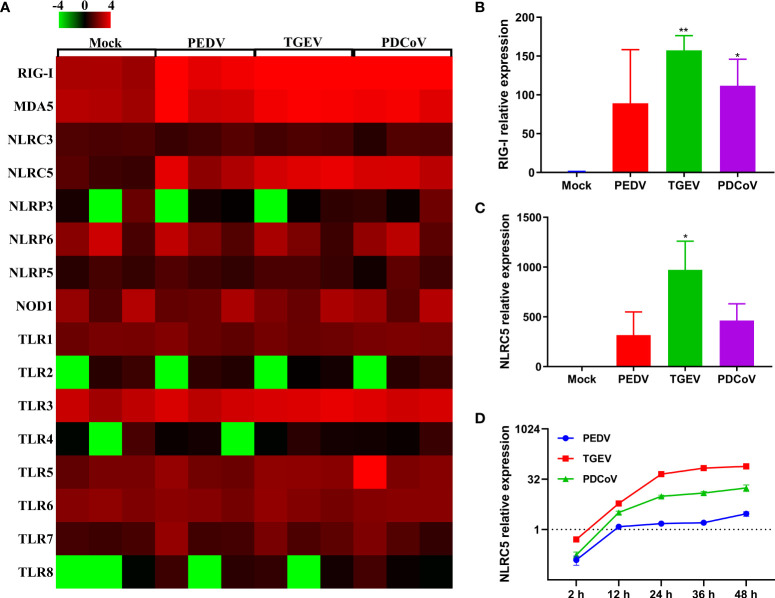
The differential ability of SECoVs to manipulate PRRs and NLRs expression in enteroids. **(A)** Heat map of PRRs and NLRs expression in jejunal enteroids infected SECoVs. Total RNA was extracted from enteroids after infection with or without PEDV, TGEV, or PDCoV for 24 h, and the transcriptional profiles of PRRs and NLRs were determined by RNA-Seq. Data are based on log (RPKM) values (n = 3). (B-C). Verification of RIG-I and NLRC5 expression in jejunal enteroids by RT-qPCR. Monolayers of jejunal enteroids were infected with PEDV (MOI = 5), TGEV (MOI = 1), or PDCoV (MOI = 2) for 24 h, and the mRNA levels of RIG-I **(B)** and NLRC5 **(C)** were detected by RT-qPCR. Data represent the means ± SEMs (n = 3). The differences between groups were compared using the Student’s *t* test. Error bars denote the deviations, and the *p* values are expressed as follows: *p <0.05, and **p <0.01. **(D)** The expression kinetics of NLRC5 in jejunal enteroids after SECoVs infection. Following PEDV, TGEV, or PDCoV infection, jejunal enteroid monolayers were collected at the indicated time points for the quantification of NLRC5 expression by RT-qPCR. The scale data of panel D were taken as log_2_ (fold change) values.

The initiation of cellular responses to viral infection requires the recognition of viral genomes and proteins by various host pattern recognition receptors (PRRs) and nucleotide-binding oligomerization domain (NOD)-like receptors (NLRs), thus leading to multiple productions of type I and III IFNs to eliminate invading viruses (31, 32). To explore whether the expression of PRRs and NLRs determines the differential ability of SECoVs to manipulate the IFN responses, we investigated the transcriptional expression of PRRs and NLRs in enteroids following SECoVs infection, including RIG-I-like receptors (RLRs) and toll-like receptors (TLRs), which are mainly responsible for sensing RNA virus infection (33), as well as NLRs, which largely mediate the activation of rapid inflammatory responses and restriction of pathogen replication (34). The heat map results showed that the expression profiles of PRRs and NLRs in enteroids induced by the three SECoVs were distinct, but the overall expression trend of these genes was similar (**Figure 7A**). In line with the induction of IFN responses, the transcriptional levels of RLRs members in enteroids, including RIG-I and melanoma differentiation-associated gene 5 (MDA5) that that are important for inducing IFNs production in intestinal epithelia (35), were largely upregulated by TGEV, followed by PDCoV (**Figure 7A**). In contrast to TGEV and PDCoV, PEDV infection induced a much lower expression level of RIG-I and MDA5 (**Figure 7A**). Additionally, we observed that TLRs expressions in enteroids were nearly unchanged except that TLR8 expression was increased after SECoVs infection (**Figure 7A**). Notably, the transcriptional level of NLRC5, one of the NLRs members, was markedly upregulated by all the three SECoVs, and the upregulation pattern of NLRC5 was in line with the expression of RLRs (**Figure 7A**), suggesting that the differential expression of PRRs and NLRs in enteroids is closely related with the diverse ability of SECoVs to mediate the IFN responses. Consistent with the dataset of RNA-Seq, the expression levels of RIG-I and NLRC5 were significantly increased in enteroids after the three SECoVs infections, and TGEV infection elicited the highest elevation of RIG-I and NLRC5, followed by PDCoV and PEDV (**Figures 7B, C**). Moreover, the expression kinetics of NLRC5 in jejunal enteroids following SECoVs infection demonstrated that compared with mock-infected control, the expression of NLRC5 at 48 hpi was greatly increased by 73-fold and 17-fold in TGEV- and PDCoV-infected enteroids, respectively; whereas PEDV infection only increased NLRC5 expression about 3-fold at 36 hpi (**Figure 7D**). In all, these observations suggest that the differential expression of PRRs and NLRs in enteroids highly correlates with the different induction of IFN responses by the three SECoVs.

### Differential Induction of SLA-I and Its Associated Antigen-Presentation Genes Expression in Enteroids Following SECoVs Infection

**Figure 8:**
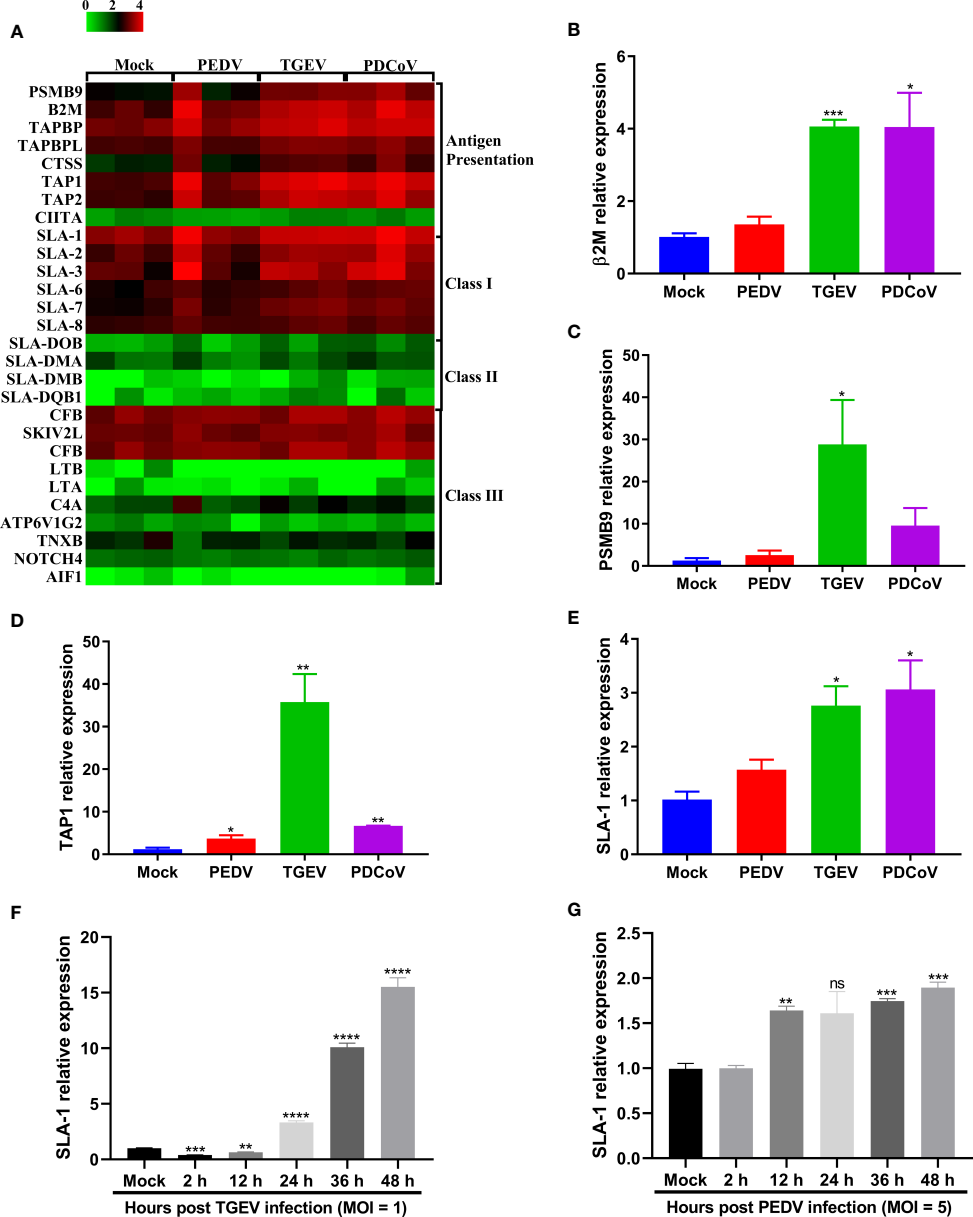
TGEV and PDCoV, not PEDV, robustly upregulate the expression of SLA-I and its associated antigen-presentation genes expression in enteroids. **(A)** Heat map of SLA molecules and genes associated with antigen-presentation in enteroids following infection with SECoVs. The transcriptional profiles of SLA molecules (SLA-I, SLA-II, and SLA-III) and genes associated with antigen-presentation in enteroids following infection with PEDV, TGEV, and PDCoV were determined by RNA-Seq. Data are based on log(RPKM) values (n = 3). **(B–E)** Verification of the expression of SLA-I and it’s associated with antigen-presentation by RT-qPCR. Jejunal enteroid monolayers were mock-infected or infected with PEDV, TGEV, or PDCoV for 24 h. The mRNA expressions of β2M **(B)**, PSMB9 **(C)**, TAP1 **(D)**, and SLA-1 **(E)** were measured by RT-qPCR. **(F, G)** The expression kinetics of SLA-1 in jejunal enteroids induced by TGEV **(F)** and PEDV **(G)** were assessed by RT-qPCR. The differences between groups were compared using the Student’s *t* test. Error bars denote the deviations, and the *p* values are expressed as follows: *p < 0.05, **p < 0.01, ***p < 0.005, and ****p < 0.001. ns, not significant.

MHC-I and MHC-II molecules play a critical role in the control and clearance of viral infections by presenting peptides to CD8^+^ and CD4^+^ T cells, respectively, and further leading to the initiation of an adaptive immune response (36, 37). Emerging evidence shows that T cell immunity is critical for the host to efficiently control coronaviruses infection, such as severe acute respiratory syndrome coronavirus 2 (SARS-CoV-2) (38, 39), while the interactions between T cell immunity and SECoVs infection remain unclear. To elucidate whether SECoVs infection would manipulate T cell immune responses, we compared the transcriptional expression of both SLA molecules and other components associated with antigen presentation in jejunal enteroids following PEDV, TGEV, and PDCoV infection. Compared with mock-infected control, we observed a broad upregulation of antigen-presentation molecules following both TGEV and PDCoV infection, especially genes involved in SLA-I antigen processing and presentation, such as proteasome subunit beta 9 (PSMB9), β2-microglobulin (β2M), and transporter associated with antigen processing (TAP1, TAP2, TAPBP, and TAPBPL); in contrast, PEDV infection resulted in a minimal increase of these genes (**Figure 8A**). Furthermore, both TGEV and PDCoV infection strongly increased expression of classic SLA-I family members, including SLA-1, SLA-2, and SLA-3, whereas PEDV infection induced a lower expression level of these genes than TGEV and PDCoV (**Figure 8A**). Interestingly, all the three SECoVs infections had no impact on the expression of SLA-II and SLA-III molecules (**Figure 8A**). The mRNA expression levels of several typical SLA-I and antigen-presentation genes were further confirmed by RT-qPCR, which showed a similar trend as observed in RNA-Seq (**Figures 8B**–**E**). Similarly, the expression kinetics of SLA-1 in SECoVs-infected jejunal enteroids revealed that SLA-1 expression was notably elevated by 3-fold to 39-fold from 24 hpi to 48 hpi after TGEV infection; in contrast, PEDV infection only slightly increased SLA-1 expression (**Figures 8F, G**). Together, these data indicate that SECoVs infection modulates the expression of SLA-I and its associated antigen-presentation genes instead of SLA-II and SLA-III molecules, especially during TGEV and PDCoV infection.

**Figure 9:**
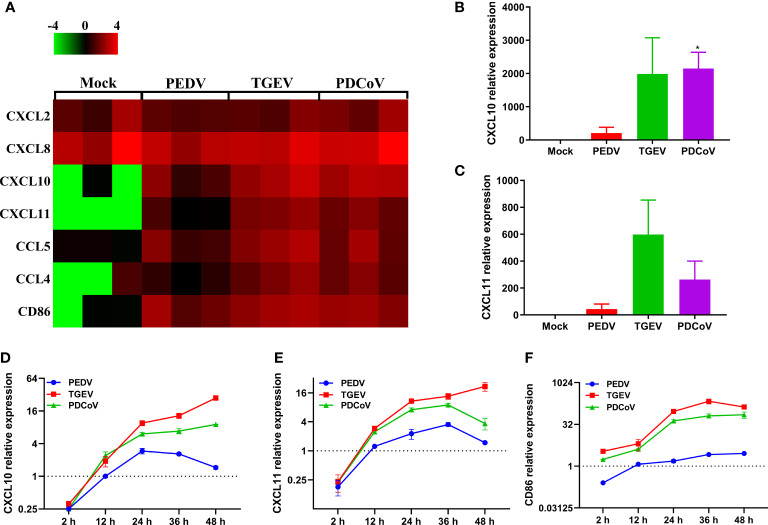
TGEV and PDCoV, not PEDV, strongly promote the expression of chemokines related to T cell immunity in enteroids. **(A)** Heat map of significant induction of chemokines related with T cell immunity in enteroids infected with SECoVs. Monolayers of jejunal enteroids infected with PEDV, TGEV, and PDCoV were harvested at 24 hpi. The transcriptional expression of chemokines in enteroids was confirmed by RNA-Seq. Data are based on log(RPKM) values (n = 3). **(B, C)** Jejunal enteroids monolayers were infected with PEDV, TGEV, and PDCoV, and collected at 24 hpi to analyze the expression of CXCL10 **(B)**, and CXCL11 **(C)** by RT-qPCR. **(D–F)**. Following infection with PEDV, TGEV, and PDCoV, jejunal enteroid were collected at the indicated time points. The expression kinetics of CXCL10 **(D)**, CXCL11 **(E)**, and CD86 **(F)** were quantified by RT-qPCR. Data are presented as the means ± SEMs (n = 3). The scale data of panel D-F were taken as log_2_ (fold change) values. The differences between groups were compared using the Student’s *t* test. Error bars denote the deviations, and the *p* values are expressed as follows: *p < 0.05.

Chemokines, as a bridge between innate immunity and adaptive immunity by recruiting T cells to the infection local sites, play vital roles in the host defense against virus infection (40, 41). By comparing the transcriptional profiles of several classic chemokines, we observed that SECoVs infection resulted in a significant expression of chemokines, especially CXCL10, CXCL11, CCL5, and CCL4 (**Figure 9A**), which are crucial for recruiting T cells (42, 43). Furthermore, we found that CD86 that provides necessary costimulatory signals for T cell activation and survival (44), was substantially upregulated after SECoVs infection (**Figure 9A**). Globally, the induction of these genes by TGEV and PDCoV was superior to PEDV (**Figure 9A**). The RNA-Seq results of CXCL10 and CXCL11 were further verified by RT-qPCR (**Figures 9B, C**). Similarly, the expression kinetics of CXCL10, CXCL11, and CD86 in jejunal enteroids following SECoVs infection showed that compared with mock-infected control, both TGEV and PDCoV infection robustly induced the expression of CXCL10, CXCL11, and CD86, and TGEV infection induced the highest levels of these genes, followed by PDCoV infection; whereas PEDV infection only induced a minor increase of these genes (**Figures 9D**–**F**), suggesting a common mechanism may be utilized by TGEV and PDCoV to modulate T cell immunity. In summary, these data indicate that SECoVs infection leads to a remarkable upregulation of antigen presentation and chemokines related to T cell immunity in a virus-specific manner, demonstrating that SECoVs infection exerts a differential role in modulating T cell immunity of intestinal epithelia.

### IFNs Stimulation Contributes to the Induction of SLA-I and Its Associated Antigen-Presentation Genes

**Figure 10:**
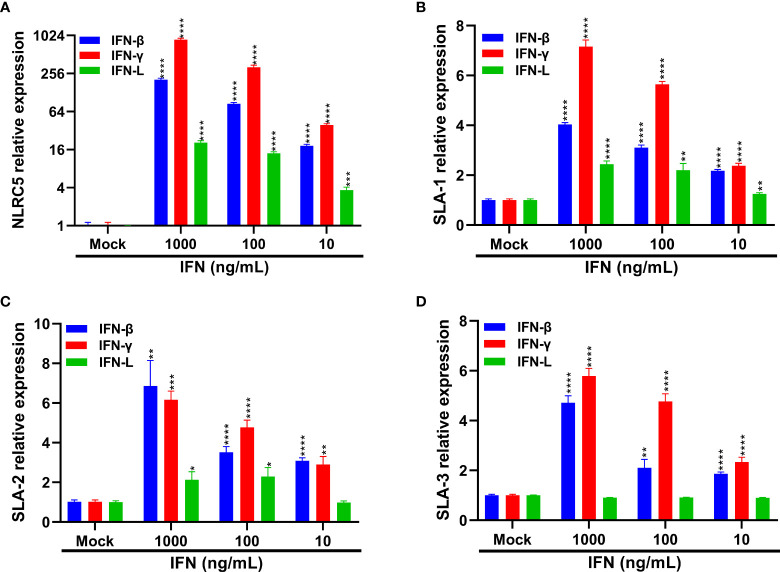
IFNs treatment increases the expression of SLA-I and its associated antigen-presentation genes. **(A–D)** IPEC-J2 cells were respectively pretreated with IFN-β, IFN-γ, and IFN-L at the indicated concentrations for 24 h. The expression levels of NLRC5 **(A)**, SLA-1 **(B)**, SLA-2 **(C)**, and SLA-3 **(D)** were examined by RT-qPCR. Data are presented as the means ± SEMs (n = 3). The scale data of panel **(A)** were taken as log_2_ (fold change) values. The differences between groups were compared using the Student’s *t* test. Error bars denote the deviations, and the *p* values are expressed as follows: *p < 0.05, **p < 0.01, ***p < 0.001, and ****p < 0.0001.

Previous studies have reported that type I and II IFNs can enhance the expression of MHC-I molecules and NLRC5, an important modulator of MHC-I antigen presentation, thus leading to the induction of MHC-I molecules (45–48). IFN-L instead of type I IFN largely accounts for the superior MHC-I expression in thymic epithelial cells (49). As we demonstrated above that SECoVs infection elicited a robust production of NLRC5 and type I and III IFNs (**Figures 5**, **7**), it is rational to hypothesize that IFNs are contributing factors for MHC-I induction during SECoVs infection. To assess this speculation, we first detected the mRNA expression of NLCR5 in the intestinal porcine epithelial cell line J2 (IPEC-J2) following stimulation with IFNs at the indicated concentrations for 24 h, including IFN-β and IFN-L. As expected, the positive control IFN-γ induced robust expression of NLRC5 and SLA-I molecules (**Figure 10**). As previously described (45, 47), we found that IFN-β treatment significantly induced NLRC5 expression in a dose-dependent manner, while IFN-L stimulation only led to a minor alteration (**Figure 10A**). Consistent with the expression pattern of NLCR5, we observed that IFN-β treatment substantially increased the expression of SLA-1, SLA-2, and SLA-3; in contrast, IFN-L treatment exerted a limited effect on the induction of SLA-I molecules (**Figures 10B**–**D**), suggesting that type I IFN instead of type III IFN largely mediates the expression of MHC-I molecules through the induction of NLRC5 during SECoVs infection. In a word, these results reveal that IFN signaling, particularly type I IFN, plays a vital role in the modulation of MHC-I antigen presentation in intestinal epithelia, indicating that SECoVs-induced IFNs may involve in the modulation of T cell responses.

## Discussion

Swine enteric coronaviruses (SECoVs) account for the majority of lethal watery diarrhea in neonatal pigs and pose significant economic and public health burdens (1, 2, 50). A systematic and comprehensive understanding of the host cellular responses to different SECoVs infection may elucidate different virulence seen among SECoVs and could catalyze novel drug development efforts to combat swine enteric coronavirus disease (SECD). Here, we employed primary porcine enteroids derived from intestinal crypt stem cells as SECoVs infection model that recapitulate the cellular composition and structure of intestinal epithelia and performed the whole transcriptome in jejunal enteroids after infection with three major SECoVs (PEDV, TGEV, and PDCoV). We revealed that TGEV and PDCoV infections resulted in the strong upregulation of both IFN responses and antigen-presentation gene expressions. In contrast, the virulent PEDV failed to robustly modulate the IFN responses and ISG induction, as well as the activation of antigen-presentation gene expression. Meanwhile, we showed that IFNs stimulation markedly increased the expression of major histocompatibility complex class I (MHC-I) molecules [known as the swine leukocyte antigen class I (SLA-I) in pigs]. Altogether, this study demonstrates a contrasting, similar, and unique mechanism exploited by SECoVs to modulate the global IFN responses and the expression of antigen-presentation-associated genes.

SECoVs primarily infect the small intestine (ileum and jejunum) *in vivo*, and cause similar clinical symptoms, thus a better understanding the interplay between SECoVs and host intestinal epithelium can shed light on the similarities and differences of viral pathogenesis. RNA sequencing (RNA-Seq) is a powerful tool to explore the host cellular response to viral infections. Although others have investigated the host cell responses to SECoVs infection in nonporcine or nontransformed cell lines by RNA-Seq, such as Vero cells, PK-15 cells, and IPEC-J2 cells (24, 51–53), which may have some limitations as these cell lines cannot recapitulate the transcriptional signatures exhibited by the specific small intestinal segments following the three SECoVs infection. We previously uncovered that primary porcine enteroids maintain inherited segmental variation of the intestinal epithelium and can model the tissue tropism of SECoVs infection *in vivo*, highlighting that enteroids are a more physiologically relevant cell culture model of intestinal epithelia (18, 20). These findings demonstrate that porcine enteroids are a unique system by which to study the interactions between SECoVs and host intestinal epithelia, which can better recapitulate the events associated with SECoVs infections *in vivo.*

Upon viral infection, mammalian hosts launch an immediate innate immune response, including the production of IFNs, particular type I IFN (IFN-α/β) and type III IFN-lambda (IFN-L), which further bind to their corresponding surface heterodimeric receptors (IFNAR for type I IFN; IFNLR1 and IL10RB for IFN-L), leading to the activation of the Janus kinase 1 (JAK1)-signal transducer and activator of transcription 1/2 (STAT1/2) pathways, thus subsequently leading to a variety of ISGs expression to control invading viruses (14, 25, 26, 30, 54). Previous studies have shown that PEDV infection inhibits type I IFN production in several cell types including Vero cells, MARC-145, and porcine intestinal epithelial cells (IECs) (15–17). In contrast, despite both PEDV and TGEV belonging to alphacoronavirus, infection of TGEV *in vitro* and *in vivo* robustly induces high levels of type I IFN production (19, 55, 56). The IFN induction by PDCoV infection is divergent and is related to the cell types (18, 57–59). Consistent with previous results (18, 19), we found that TGEV infection notably induced the IFN and ISG responses in jejunal enteroids, followed by PDCoV infection, but a much lesser extent of IFN and ISG responses was observed in PEDV-infected enteroids compared with the other two SECoVs (**Figures 5**, **6**). Interestingly, we revealed that although the ability of TGEV and PDCoV to activate the IFN responses was superior to PEDV, both TGEV and PDCoV replicated more robustly than PEDV at the early stage of infection in jejunal enteroids (**Figures 1**, **5**, and **6**), suggesting that, unlike PEDV, TGEV and PDCoV may employ some different strategies to evade the IFN responses instead of inhibiting the induction of IFNs. Actually, our previous observation showed that TGEV antagonizes the IFN responses by upregulating the negative regulator suppressor of cytokine signaling (SOCS1/3) expression rather than reducing IFN production (60). It is worthwhile to further elucidate the underlying specific mechanisms escaping the IFN responses during various SECoVs infections in intestinal epithelia. The antiviral IFNs are triggered by the PRRs especially RIG-I and MDA5 sensing of invading RNA viruses (31, 32). Consistent with this, the expression levels of RIG-I and MDA5 in enteroids highly corresponded with the expression patterns of IFN and ISG responses (**Figure 7**). Globally, both TGEV and PDCoV infection in jejunal enteroids elicited substantial upregulation of RIG-I and MDA5 expression although the induction of RIG-I and MDA5 by PDCoV was slightly inferior to TGEV; while PEDV infection only caused a minimal transcriptional variability of PRRs (**Figure 7**), indicating that the differential activation of PRRs expression is closely related to the ability of SECoVs to modulate the IFN responses.

Emerging evidence demonstrates that CD8^+^ T cells efficiently contribute to the clearance of coronaviruses in the host (38, 39, 61). MHC-I and its associated antigen presentation genes are critical for the initiation of CD8^+^ T cell immunity (36, 37). For SECoVs infections, information remains extremely limited regarding the initiation and importance of CD8^+^ T cell immunity in the content of SECoVs infection. We first demonstrated the distinct expression profiles of SLA-I molecules and their associated antigen presentation genes in primary intestinal enteroids following PEDV, TGEV, and PDCoV infection. TGEV and PDCoV infection prominently elevated the expression of SLA-I and its associated antigen presentation genes (PSMB9, β2M, TAP1, TAP2), whereas only minor increases were observed in PEDV-infected enteroids (**Figure 8**). Surprisingly, the infection of the three SECoVs had no obvious impact on the expression of SLA-II and SLA-III molecules (**Figure 8**), suggesting specific targeting of SLA and antigen presentation molecules by SECoVs. The potential role of intestinal epithelia as non-professional antigen-presenting cells in priming MHC-I-restricted virus-specific cytotoxic T lymphocyte (CTLs) response has not received enough attention; it deserves a deep exploration of the pervasive interactions between the host intestinal epithelia and CD8^+^ T cell responses during SECoVs infection in the future. Moreover, we unveiled that both TGEV and PDCoV infection caused a high-level expression of chemokines mainly associated with T cell immunity, such as CXCL10, CXCL11, and the important costimulatory molecule CD86, but not significant in PEDV-infected enteroids (**Figure 9**), suggesting that the infection of TGEV and PDCoV may activate a stronger T cell immunity than PEDV. In accordance with our data, the previous study demonstrated that TGEV infection results in robust virus-specific cell-mediated immune responses (62), but the underlying mechanisms exploited by different SECoVs to manipulate CD8^+^ T cell immunity and the importance of T cell immunity in controlling SECoVs infection remain unknown, which need to be further elucidated.

Previous results show that type I and II IFNs play crucial roles in promoting the expression of MHC-I associated molecules (45, 47, 48). A recent study reported that IFN-L instead of type I and II IFNs largely modulate the expression of MHC-I molecules on thymic epithelial cells (49). Consistently, we found that the expression of antigen presentation genes was correlated to the transcriptional expression patterns of type I and III IFNs after SECoVs infection (**Figures 5**, **8**). While IFN-L treatment only caused a minimal change of SLA-I molecules in enteroids (**Figure 10**), demonstrating that the induction of MHC-I molecules by IFN-L may vary among different cell types. Moreover, we demonstrated that IFNs stimulation, particularly type I IFN, significantly upregulated the expression of NLRC5 (**Figure 10**), a master regulator of MHC-I molecules (46), suggesting that SECoVs-mediated MHC-I induction may be through upregulating IFN-induced NLRC5 production. Whether SECoVs-elicited IFNs regulate SLA-I antigen-presentation and how SECoVs manipulate NLRC5 expression still need to be further explored.

In summary, this study demonstrates that porcine enteroids can be used as a valuable model to assess the host intestinal epithelial responses to SECoVs infection and highlights the utility of leveraging viral cross-comparisons as a powerful means to survey contrasts in the induction of host innate immunity and gene expression associated with antigen presentation during different SECoVs infections.

## Data Availability Statement

The data presented in the study are deposited in the National Microbiology Data Center (https://nmdc.cn) repository, and the accession number is NMDC40014591 to NMDC40014602.

## Ethics Statement

The animal study was reviewed and approved by the Laboratory Animal Ethical Committee of China Agricultural University, Beijing. Written informed consent was obtained from the owners for the participation of their animals in this study.

## Author Contributions

PL, LY, and GZ designed the study and analyzed the data. LY, XL, DH, and YL conducted experiments and acquired data. LY and PL drafted the manuscript, and all authors contributed revisions. All authors contributed to the article and approved the submitted version.

## Funding

Support for this work was provided by the grants from the National Key Research and Development Program of China (2021YFD1801104), the National Natural Science Foundation of China (32172865), and the Chinese Universities Scientific Fund (2021TC115).

## Author Disclaimer

The content is solely the responsibility of the authors and does not necessarily represent the official views of the funding resources.

## Conflict of Interest

The authors declare that the research was conducted in the absence of any commercial or financial relationships that could be construed as a potential conflict of interest.

## Publisher’s Note

All claims expressed in this article are solely those of the authors and do not necessarily represent those of their affiliated organizations, or those of the publisher, the editors and the reviewers. Any product that may be evaluated in this article, or claim that may be made by its manufacturer, is not guaranteed or endorsed by the publisher.
